# Diagnosis of Fanconi Anemia: Chromosomal Breakage Analysis

**DOI:** 10.1155/2012/238731

**Published:** 2012-05-24

**Authors:** Anneke B. Oostra, Aggie W. M. Nieuwint, Hans Joenje, Johan P. de Winter

**Affiliations:** Department of Clinical Genetics, VU University Medical Center, Van der Boechorststraat 7, 1081 BT Amsterdam, The Netherlands

## Abstract

Fanconi anemia (FA) is a rare inherited syndrome with diverse clinical symptoms including developmental defects, short stature, bone marrow failure, and a high risk of malignancies. Fifteen genetic subtypes have been distinguished so far. The mode of inheritance for all subtypes is autosomal recessive, except for FA-B, which is X-linked. Cells derived from FA patients are—by definition—hypersensitive to DNA cross-linking agents, such as mitomycin C, diepoxybutane, or cisplatinum, which becomes manifest as excessive growth inhibition, cell cycle arrest, and chromosomal breakage upon cellular exposure to these drugs. Here we provide a detailed laboratory protocol for the accurate assessment of the FA diagnosis as based on mitomycin C-induced chromosomal breakage analysis in whole-blood cultures. The method also enables a quantitative estimate of the degree of mosaicism in the lymphocyte compartment of the patient.

## 1. Introduction

Fanconi anemia (FA) is a cancer-prone chromosomal instability disorder with diverse clinical symptoms (Table 1) [1]. Because of its rarity and variable presentation FA may be heavily underdiagnosed [2, 3]. Clinical suspicion of FA is mostly based on growth retardation and congenital defects in combination with life-threatening bone marrow failure (thrombocytopenia and later pancytopenia), which usually starts between 5 and 10 years of age. However, the clinical manifestations are highly variable, while some of the symptoms may overlap with those observed in other syndromes, making a reliable diagnosis on the basis of clinical features virtually impossible (Table 1). Even patients presenting with a number of “typical” FA symptoms may not be suffering from FA. Cells derived from true FA patients must exhibit a hypersensitivity to chromosomal breakage induced by DNA cross-linking agents such as mitomycin C (MMC), diepoxybutane (DEB), or cisplatinum.

Indications to test for FA are typical congenital abnormalities with/without thrombocytopenia and/or marrow failure. However, congenital abnormalities may be absent, while isolated thrombocytopenia may be the only presenting symptom. In all children with aplastic anemia FA should be tested as the possible underlying disease. In children and adults with cancer and an unusual response to DNA-damaging agents such as chemotherapy or radiotherapy (severe skin reactions or mucositis, longlasting aplasia), FA should also be tested for. Similarly, in adults with carcinomas (typically located in the mouth/esophagus or anogenital region) at relatively young age, FA should be considered. Cancer or leukemia may be the first symptom of FA, while congenital abnormalities and marrow failure may be absent altogether, the latter especially in cases with hematopoietic mosaicism [4–6].

The cellular phenotype typical for FA is ascertained using phytohaemagglutinin-stimulated whole-blood (T lymphocyte) cultures. Although it has been considered the gold standard for diagnosing FA, the test is not 100% specific. A few cases of Nijmegen breakage syndrome have been reported to give a false positive result [7–9], which can be excluded by screening the *NBS1* gene for mutations. In addition, patients suffering from the cohesinopathies Roberts syndrome (mutated in *ESCO2*) and Warsaw breakage syndrome (mutated in *DDX11*) may score positive in the test [10]. Additional “atypical FA” or “FA-like” patients have been reported as case reports [11, 12]. Somewhat controversially, the “FA-like” patient found to be mutated in *RAD51C* has been assigned to a distinct genetic FA subtype (FA-O) [13].

Approximately 80% of the patients referred for FA diagnostic testing because of bone marrow failure score negative in the chromosomal breakage test. These “true negatives” have other causes of marrow failure and most often represent cases with acquired aplastic anemia.

Lymphocyte mosaicism occurs in a sizable proportion of FA patients (estimated at 10–30%) and is caused by spontaneous genetic reversion at the disease locus in hematopoietic progenitor cells; the reverted cells may (partially) correct the bone marrow failure [14–18]. In most of these cases FA can still be diagnosed by testing peripheral blood, since a portion of the cells will still show hypersensitivity to cross-linking agents. Occasionally, the percentage of reverted cells has reached such a high level as to produce a false negative diagnosis. In such cases cross-linker sensitivity may be tested in skin fibroblasts, which are not known to be affected by mosaicism. After a positive breakage test result has been obtained, screening for mutations in the known FA genes is warranted.

Laboratory studies have revealed as many as 15 distinct “complementation groups” or genetic subtypes: FA-A, -B, -C, -D1, -D2, -E, -F, -G, -I, -J, -L, -M, -N, -O, and -P [13, 19–21]. For all subtypes known to date the disease genes have been identified. Global relative prevalences are difficult to estimate, as the values may differ considerably depending on the ethnic background, due to founder effects. All FA genes are localized on autosomes, except *FANCB*, which is X-linked and subject to X inactivation in female carriers [22]. These two different modes of inheritance have important consequences for the counseling of FA families.

Recognition of FA as a chromosomal instability disorder was originally based on chromatid-type aberrations spontaneously occurring in standard cytogenetic preparations. However, this phenomenon was later found to be highly variable and considered not reliable for diagnostic purposes. After the discovery of an extreme sensitivity of FA cells to the chromosome-breaking effect of the cross-linking agents mitomycin C (MMC) [23] and diepoxybutane (DEB) [24], this feature has become routinely utilized to diagnose FA by a “chromosomal breakage test.” In this test, T lymphocytes in a peripheral blood sample are cultured in the presence of a cross-linking agent, after which chromosomal aberrations are quantified in metaphase spreads. Numerous variations of the test are used in the various cytogenetic laboratories, with significant differences in exposure times and drug concentrations. Also, the ways in which data are evaluated are diverse. We have encountered opposite conclusions from different laboratories based on the very same primary data set, due to a lack of experience in performing the test and evaluating the resulting data. Evidently, there is a great need for a clearly described reliable protocol for the accurate diagnosis of FA patients.

## 2. Methods and Results

Here we describe a laboratory protocol that has evolved during 30 years of experience and which we can recommend for the unambiguous diagnosis of the vast majority of FA patients, including patients with hematopoietic mosaicism. The test is based on the 72 hour whole-blood cultures as routinely applied in cytogenetics laboratories to make chromosomal preparations for karyotypic analysis. Metaphase spreads are Giemsa-stained (not banded) and analyzed for microscopically visible chromatid-type aberrations. For technical details the reader is referred to the appendices. Laboratories that are not set up to do this type of assay or have no experience with diagnosing FA on a regular basis should be advised to refer to a laboratory where the test is applied on a routine basis, rather than attempting to carry out a “similar” test that is considered a plausible alternative. The test might be omitted if a proband belongs to an ethnic population with a high carrier frequency for a specific FA gene mutation. Demonstrating this mutation in the proband would be diagnostic for FA, although the result may not provide information about possible mosaicism.

## 3. Discussion

It should be pointed out that, even though we have chosen to use MMC as the cross-linking agent, DEB is used in a widely accepted alternative protocol [1, 26–28]. Pros and cons for using the various cross-linking agents are further discussed in the appendices.

Cell cycle analysis via flow cytometry has been used as an alternative way to diagnose FA in skin fibroblasts [29], amniocytes [30], and peripheral blood mononuclear cells [31–34]. This test is based on the fact that cells from FA patients are hypersensitive towards DNA cross-linking agents and tend to be delayed and arrested with a 4c DNA content in the late S/early G2 phase of the cell cycle [35–38]. With the exception of overt leukemia and complete lymphocyte mosaicism, the cell cycle test reliably differentiates between FA and non-FA blood samples, including non-FA patients with aplastic anemia, Nijmegen breakage syndrome, Roberts syndrome, Baller-Gerold syndrome, VACTERL, and other thrombo- and erythropenia syndromes, as these conditions lack elevated G2-phase cell fractions [39]. For details of the cell cycle assay, readers are referred to the published protocols [39, 40].

 FANCD2 western blotting is another alternative procedure to diagnose FA [40]. With this method stimulated T lymphocytes are tested for the occurrence of the ubiquitinated isoform of FANCD2, which readily reveals FA in cases where this isoform is lacking (subtypes A, B, C, D2, E, F, G, I, L, and M). This is a convenient alternative for diagnosing >90% of all FA patients. A disadvantage is that the subtypes with a defect downstream of FANCD2 ubiquitination (D1, J, M, N, O, P and possibly new subtypes) are not diagnosed as FA. In addition, true FA cases with significant lymphocyte mosaicism may also be missed by FANCD2 western blotting.

Why would a relatively laborious breakage test still be relevant now that next-generation sequencing (NGS) is available to determine mutations in FA genes? Two types of result from NGS would require assessment of the cross-linker sensitive cellular phenotype. First, unclassified sequence variations may be identified, whose pathogenic status remains uncertain until functionally tested. Second, if all known FA genes were found to be unaffected by mutations, a putative new FA gene may be found mutated. Proof of identity as a new FA gene requires the demonstration of cellular hypersensitivity to cross-linking agents and some form of functional test where introduction of a wild-type allele should correct the phenotype.

## Figures and Tables

**Figure 1:**
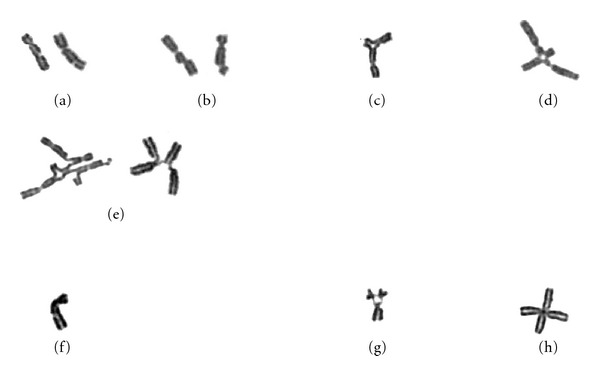
Examples of chromosomal aberrations typically observed in a MMC-induced chromosomal breakage assay to diagnose FA. (a) Chromatid gap (broken piece in place); (b) chromatid break (broken piece dislocated); (c) chromatid interchange figure (“triradial”); (d) chromatid interchange figure (“quadriradial”); (e) other chromatid interchange figures. In the eventual analysis, (a) and (b) are counted as one, (c) and (d) as two break events. The left figure in (e) is counted as 8 break events (5 centromeres plus 3 open breaks); the right figure is equivalent to a quadriradial as in (d) (2 break events), in which two break points remained disconnected. (f), (g), and (h), are examples of nonconvincing “aberrations” that should be ignored in the analysis. (f) A gap that is not 100% convincing and should be ignored. (g) An association of 3 acrocentric chromosomes showing “satellite association”, not to be confused with a triradial, as in (c). (h) Two overlapping chromosomes, not to be confused with a true quadriradial, as in (d).

**Figure 2:**
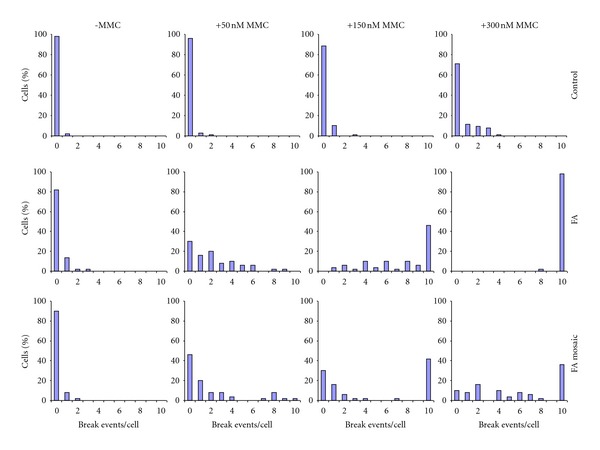
Evaluation of MMC-induced chromosomal breakage in stimulated T lymphocyte cultures. Upper row: healthy control; middle row: FA patient; lower row: mosaic FA patient. The healthy control shows breakage only at 300 nM, where the FA patient shows massive breakage (no normal cells present). Mosaicism is evident from the two highest concentrations of MMC, where there are still normal cells present next to cells showing an FA-like breakage rate (>10 breaks/cell). A crude estimate of the proportion of reverted T cells in this mosaic patient would be ~40%.

**Table 1:** General features and symptoms associated with Fanconi anemia.

Birth prevalence	0.5–2.5 per 10^5^ newborns; varies with ethnic background.

Mode of inheritance	Autosomal recessive (>98%) and X-linked (~1-2%).

Carrier frequency	Traditional overall estimate: “1/300 worldwide.” Needs reassessment according to subtype and ethnic background.

Congenital abnormalities*	Radial ray abnormalities (aplastic or hypoplastic radii and absent or extra thumbs) and other skeletal abnormalities; small head circumference; abnormal shape of the ears;** microphthalmia**; ectopic or horse-shoe kidney; **hypogonadism**; heart abnormalities; intestinal or anal atresia.

Other somatic abnormalities*	**Short stature/retarded growth; reduced fertility; skin pigmentation abnormalities (hyperpigmentation, café-au-lait spots)**; deafness. Endocrinopathy affecting the pancreas (diabetes mellitus), growth hormone deficiency, and hypothyroidism; early menopause.

Hematological symptoms	Bone marrow failure or aplastic anemia typically starting at 5–10 years with thrombocytopenia. Exception: D1 and N patients may die before that age from AML or other childhood solid tumors (such as medullo- or nephroblastoma).

Cancer risk	800-fold increased risk of AML, mostly occurring at age 5–15 years, typically after the onset of marrow failure. At older ages there is a similarly increased risk of solid tumors, mainly carcinomas of the head and neck or oesophagus, as well as, in females, the vulva and vagina. D1 and N patients typically develop malignancies during early childhood (<5 years).

Overlapping syndromes**	*Inherited bone marrow failure syndromes*: Dyskeratosis congenita, Diamond-Blackfan anemia, Shwachman-Diamond syndrome, severe congenital neutropenia, thrombocytopenia absent radii (TAR) syndrome, amegakaryocytic thrombocytopenia.*Other overlapping syndromes*: Baller-Gerold syndrome, *Nijmegen breakage syndrome*, Rothmund-Thomson syndrome, *Roberts syndrome*, *Warsaw Breakage syndrome*, DK-phocomelia, VACTERL hydrocephalus syndrome, Wiskott-Aldrich syndrome.

*Many symptoms show highly variable penetrance. In a sizable proportion of patients (ca. 30%), congenital abnormalities may be absent altogether. Features in bold are most consistently associated with the FA phenotype.

**For an overview of the overlapping inherited bone marrow failure syndromes, see [5, 25]. For the other overlapping syndromes, the reader is referred to the OMIM database. Three overlapping syndromes may score positive in a chromosomal breakage test (italic): Nijmegen breakage syndrome [7–9], Roberts syndrome, and Warsaw Breakage Syndrome [10].

## Appendices

### A. Laboratory Protocol for Testing MMC-Induced Chromosomal Breakage

#### A.1. Materials

1. Heparinized venous blood (≥2 mL; preferably freshly drawn, or kept at room temperature for no longer than 48 h) from the patient to be tested and from a healthy control.
2. RPMI or Ham's F10 culture medium, including 15% fetal bovine serum, streptomycin, penicillin, and phytohemagglutinin, as utilized in standard cytogenetic whole-blood cultures.
3. Mitomycin C (MMC, mol. wt. 334.33, Kyowa Hakko Kogyo Co., Ltd., Tokyo, Japan, clinical grade), available in vials of 2 mg with 48 mg NaCl, to be stored at 4°C.
4. Materials for the preparation of metaphase spreads.

#### A.2. Culturing and Cytogenetics Methods

1. Prepare a stock solution of MMC at 1.5 mM (0.5 mg/mL) by adding 4 mL sterile water per vial; this solution is stable for 3 months at 4°C. It is mandatory “*not*” to freeze the MMC stock solution, since—upon thawing—an unknown quantity of MMC appears as crystals that do not readily redissolve.
2. Prepare whole-blood cultures from the patient and the healthy control, as usual for a standard cytogenetic analysis [25]. You need 4 cultures for the patient and 4 for the healthy control, who should *not* be a brother or sister of the patient. Initiate the cultures by adding 0.5 mL blood to 4.5 mL complete medium.
3. Add, at the time of culture initiation, to each set of 4 cultures: 0, 50, 150, and 300 nM MMC, as indicated below.
  - Dilute 1 part stock solution plus 9 parts H_2_O →*solution A* (150 *μ*M).
  - Dilute 1 part solution A plus 4 parts H_2_O →*solution B* (30 *μ*M).
  - Add to the 4 cultures from each individual:
    - 50 *μ*L saline → final concentration: 0 nM,
    - 8.3 *μ*L solution B → final concentration: 50 nM (optional),
    - 25 *μ*L solution B → final concentration: 150 nM,
    - 50 *μ*L solution B → final concentration: 300 nM. N.B. If insufficient blood should be available, the 50 nM cultures may be omitted.
4. Harvest at 72 h, after colcemid treatment during the last 40 min (Sigma, demecolcine final concentration 200 ng/mL). Prepare at least 4 microscope slides for every culture; make more slides if mitotic activity is low, to end up with at least several hundreds of metaphases, accounting for the possibility that a large proportion will later be judged unacceptable for microscopic analysis. Stain with Giemsa only. Do not apply any banding technique. Store remaining suspension at –20°C, for future use, if necessary.

#### A.3. Scoring the Aberrations

It is important to realize that quantification of chromosomal aberrations shows significant differences between laboratories. From a comparative study it appeared that the most important source of disagreement was about whether particular aberrations really existed or not, and about the definition and scoring of gaps [41]. It is therefore mandatory to score metaphases from coded slides (“blind”), that is, without knowing the identity of the preparation you are scoring. Do not score more than 25 cells per slide. This is to reduce the possibility of biased scoring, which would result from inspecting too many metaphases from the same slide. To obtain sufficient statistical power of the breakage data, attempt to find and score at least 50 scorable metaphases per culture (to be scored from at least two slides).

##### A.3.1. Coding and Organizing the Slides before Scoring

After staining, divide the slides into two equal sets per culture, each set containing 2, 3, or more slides (depending on metaphase yield) to allow the analysis of 25 scorable metaphases per set (see also Appendix A.2, point 4). Cover the unique identifier information on the slide with a piece of nontransparent tape. Write a random code on each set of slides and distinguish multiple slides within a set by adding A, B, C, and so forth.

Example 1 for every culture, you end up with 4 slides or more (depending on the mitotic index), coded as follows:* [random code-1]A*, *[random code-1]B*, and so forth; *[random code-2]A*, *[random code-2]B*, and so forth. The scoring of metaphases (see below) starts with slide *[random code-1]A* until 25 metaphases have been examined. If fewer metaphases were found on the slide, proceed with slide *[random code-1]B*, and so forth, until the desired number of metaphases (in our case: 25) have been scored. Follow the same procedure for *[random code-2]A, -B, -C*, and so forth. After finishing the scoring of all preparations, the codes are uncovered and the two data sets from the various cultures are combined to provide results per 50 metaphases.

##### A.3.2. How to Score Chromosomal Aberrations

Systematically select the metaphases to be analysed: search, at 400x magnification, for metaphases judged suitable for evaluation of chromosomal integrity. To avoid a bias for relatively undamaged metaphases, do not at this stage select on the basis of “quality,” since “nice-” looking metaphases tend to have fewer aberrations. Rather, every next metaphase encountered should—in principle—be scored, unless it must be rejected because it fails to meet the observer's criteria for adequate spreading, state of condensation of the chromosomes (not too long or too short), adequate staining and morphology (clearly recognizable chromosomes with clearly visible centromeres), and adequate ploidy. When a metaphase meets these criteria, that metaphase *must* be scored, at 1000x magnification, even if “difficult” aberrations are subsequently encountered. However, be sure to score only the *really convincing* aberrations while ignoring the unconvincing ones. Distinguish the following types of aberration:

1. chromatid gap, an interruption in the staining of a chromatid 1-2 times the width of that chromatid (Figure 1(a));
2. chromatid break, where the interruption is more than 2 times the width or where the broken piece of chromatid appears dislocated, as in Figure 1(b);
3. triradial chromosome, an interchange figure presumably having resulted from the misrepair of two breaks in two distinct chromosomes (Figure 1(c));
4. quadriradial chromosome, an interchange figure resulting from the misrepair of two broken chromatids in different chromosomes (Figure 1(d));
5. Other chromatid interchange figures, such as illustrated in Figure 1(e).

Chromosome-type changes, such as dicentrics, acentric fragments, and ring chromosomes, may be recorded, but these aberrations, which are extremely rare with the protocol used, should not be included in the final analysis.

##### A.3.3. How to Record the Aberrations

The aberrations observed should be recorded with the coordinates of the metaphase, so that aberrant metaphases can be retrieved whenever considered necessary. This can be achieved manually, or with the help of an automated metaphase finder equipped with a customisable scoring sheet for the evaluation of chromosomal aberrations, such as developed by Metasystems, Altlussheim, Germany. A sheet developed for manual evaluation may be obtained from the authors, upon request.

#### A.4. Analyzing the Results

##### A.4.1. Converting Aberrations into Break Events

The ratio between gaps/breaks (“open breaks”) and interchange-type aberrations (“wrongly repaired breaks”) may vary considerably. Therefore, for the final evaluation, all aberrations are converted into “break events”, which represent the primary type of aberration in an FA cell.

Chromatid gaps or breaks are counted as single break events, tri- and quadriradials as two break events each. Other interchange figures are converted into the minimum number of breaks required for their theoretical reconstruction; in practice, this means that the number of centromeres in an interchange figure is added up to the number of open breaks/gaps, see Figure 1. To avoid spending too much time on reconstructing complex interchange figures, cells showing more than 10 break events are not further quantified and are included in a common category “≥10 breaks/cell”. Evaluate the data from a histogram, in which the percentage of cells is plotted against the number of break events/cell, as illustrated in Figure 2.

##### A.4.2. Evaluating the Results: “FA”, “Non-FA”, or “Mosaic FA”

In cultures from a typical full-fledged FA patient a substantial proportion of the cells should show chromosomal breakage already at 50 nM MMC (Figure 2). At 150 nM MMC, the majority of cells should be aberrant, while at 300 nM no undamaged cells should be left and most cells should be in the category “≥10 breaks/cell”. In contrast, cultures from the healthy control should hardly or not be affected, except at 300 nM, where typically 30% of the cells may show 1 to ≤5 break events/cell.

In cultures from FA patients with lymphocyte mosaicism, two cell populations are distinguished at 300 nM MMC, one behaving like typical FA cells, that is, showing ≥10 breaks/cell, and one behaving like healthy controls, that is, largely represented by the categories 0-, 1-, and 2-breaks/cell.

In the event of a positive result (FA or mosaic FA), all asymptomatic sibs of the patient should be tested as well, which is particularly important if the sibs are considered as potential stem cell donors. A positive result indicative of FA should immediately be evident from the histogram (Figure 2). If statistical analysis is considered necessary to “prove” a dubious diagnosis, the diagnosis “FA” is likely to be wrong.

If the result indicates “non-FA”, an important question is whether the MMC concentration was correct. This is another reason why the highest concentration (300 nM) is included, since at this concentration the healthy control should show significantly elevated breakage. The difference between treated and untreated control cultures may be tested by comparing the percentages of aberrant metaphases, using a 2-sample Chi-square test. If the healthy control should fail to show a clear response to the MMC at 300 nM, the result “non-FA” is inconclusive and the test should be repeated.

### B. Technical Notes and Comments

#### B.1. Breaks and Gaps

In the 1980s the distinction between chromatid gaps and breaks has been the subject of much discussion, the issue being whether a gap represented a true double-stranded break in the DNA of a chromatid. A problem during the evaluation of aberrations is to decide which gap-like feature should be scored as a true aberration. A consensus was reached by accepting an aberration as a gap if the discontinuity in the staining of a chromatid is at least as wide as the width of the chromatid. If wider than twice the width of the chromatid, the aberration may be scored as a break [42]. If the “broken” piece appears dislocated the aberration is always scored as a break. If the interruption is considered doubtful, it should be ignored (Figure 1); this holds for all other aberrations that appear not entirely convincing.

The main reason to distinguish between chromatid gaps and breaks is that their biological impact may be different; conclusions based on significant differences in the frequency of gaps only, should be viewed with caution.

#### B.2. Saving Time on the Breakage Test

##### B.2.1. Finding Metaphases

In cases of low mitotic activity considerable time may be gained by utilizing a metaphase finding apparatus, which can perform unattended metaphase searches on multiple slides. Such apparatus may also be equipped with software for chromosomal aberration scoring, see for example, http://www.metasystems-international.com/.

##### B.2.2. Scoring Aberrations

To save time, the scoring process may be divided into phases. Score first the cultures exposed to 0 and 300 nM MMC, which may already give you an unambiguous answer.

1. The diagnosis “FA” is warranted if all cells from the proband contain multiple aberrations, whereas the majority of the control cells is normal.
2. A result *excluding* FA should be based on a modest but significantly elevated breakage level at 300 nM MMC, both in the proband and in the healthy control.

If there are too few evaluable metaphases, or if there is an indication of mosaicism, score the samples exposed to 150 nM, and—with again too few metaphases present—the 50 nM samples as well. If, however, the 300 or 150 nM cultures have provided a conclusive answer, the 50 nM cultures may be skipped.

Some laboratories score only chromatid interchanges (often referred to as “radials”) as aberrations, while ignoring chromatid breaks and gaps. Even though this is a considerable time saver, there are several disadvantages. First, with full-fledged (nonmosaic) FA patients “normal” cells (i.e., cells without interchange aberrations) are still observed at the higher MMC concentrations, leading to a false impression of mosaicism. Second, at the highest MMC concentration the aberration rate in the control does not reach statistical significance, which eliminates the internal check for drug activity. Third, chromatid interchanges are generated from chromatid breaks by an unknown joining mechanism, the precise nature of which is unclear, while variations in this process will affect the ratio between breaks and interchanges. As this ratio may vary from patient to patient, some FA patients might go unrecognised when scoring chromatid interchanges only.

##### B.2.3. High-Level Mosaicism

With the protocol described, most patients with mosaicism will be correctly diagnosed as FA, because even a minor proportion of FA-like lymphocytes will show up in the ≥10 breaks/cell category. When no FA cells can be detected in a patient with a “compelling” clinical phenotype, fibroblasts can be used to establish the diagnosis. We have encountered several FA patients whose T lymphocytes' response was indistinguishable from that in the healthy control, but whose skin fibroblasts' response clearly revealed the cellular FA phenotype (see, e.g., [6, 15]).

Breakage Test Using Fibroblasts Add MMC (50 nM) or saline to either of two 80 cm^2^ tissue culture flasks containing 1-2 × 10^6^ freshly trypsinized fibroblasts (preferably fewer than 8 *in vitro* passages) from the following individuals: (1) the patient to be tested, (2) a healthy control, and (3) a known FA patient (positive control). After 48 h at 37°C, harvest the cultures by trypsinization, following colcemid treatment for 45 min, and prepare chromosome slides. Code the slides and score for aberrations (50 cells per culture). Typical results are as follows.

*“Control (non-FA) fibroblasts”, untreated*: 3% aberrant cells (0.03 breaks/cell); *MMC-treated*: 13% aberrant cells (0.2 breaks/cell).
*“FA fibroblasts”, untreated*: 7% aberrant cells (0.07 breaks/cell); *MMC-treated*: 95% aberrant cells (7 breaks/cell).

##### B.2.4. Alternative Cross-Linking Agents

Several other DNA cross-linking agents, besides MMC, have been used to demonstrate the hypersensitive phenotype of FA cells, for example, diepoxybutane (DEB), and cis-diamminedichloroplatinum(II) (cisplatin). DEB is on the Special Health Hazard Substance List because it is a (volatile) carcinogen that should be handled with great caution. DEB is hygroscopic and—upon contact with water—slowly loses activity, with a half-life of approximately 4 days, because it hydrolyzes into 1,2,3,4-tetrahydroxybutane, a compound with no cross-linking activity. DEB is commercially available from Sigma/Aldrich. Since different batches may vary in activity, comparative testing is required, since relatively small differences may lead to incorrect conclusions regarding mosaicism in a patient (compare the standard concentration of 0.1 *μ*g/mL with 0.15 *μ*g/mL in [14]). MMC, which—as a clinically approved chemotherapeutic agent produced by Kyowa Hakko Kogyo (not by Sigma)—is under rigorous quality control and is stable when stored in the vials provided by the manufacturer. A similar argument would favour the use of cisplatin, which is also clinically approved, over DEB as the diagnostic reagent for FA. On the other hand, provided the reagents are properly handled, DEB, MMC, and cisplatin are similarly effective in establishing the FA diagnosis in a chromosomal breakage assay. According to a single comparative study, MMC appeared slightly more suitable for the assessment of lymphocyte mosaicism [14]. It should be pointed out that, unlike DEB and cisplatin, MMC requires metabolic activation in order to become active as a cross-linking agent. If metabolic activation were a variable parameter, this may be considered a disadvantage for MMC and an argument in favour of choosing cisplatin as the diagnostic cross-linker.

##### B.2.5. Conversion of Interchanges into Break Events

Although the idea of two breaks underlying each interchange between two chromosomes (often referred to as “radial”) has been considered commonplace in the genetic toxicology literature, this hypothesis is challenged by the observations of Godthelp et al. [43], who found the frequency of interchanges to increase linearly with drug dosage (rather than exponentially), implying single-hit rather than two-hit kinetics. If the single-hit principle were to be accepted, this would change the conversion factor for the quantification of interchange aberrations into break events from 2 to 1; however, adopting a conversion factor of 1 would not affect the general principle of the FA diagnosis, as described here.
